# Relating the Disease Mutation Spectrum to the Evolution of the Cystic Fibrosis Transmembrane Conductance Regulator (CFTR)

**DOI:** 10.1371/journal.pone.0042336

**Published:** 2012-08-07

**Authors:** Lavanya Rishishwar, Neha Varghese, Eishita Tyagi, Stephen C. Harvey, I. King Jordan, Nael A. McCarty

**Affiliations:** 1 School of Biology, Georgia Institute of Technology, Atlanta, Georgia, United States of America; 2 PanAmerican Bioinformatics Institute, Santa Marta, Magdalena, Colombia; 3 Department of Pediatrics, Emory University School of Medicine, Center for Cystic Fibrosis Research, Children’s Healthcare of Atlanta, Atlanta, Georgia, United States of America; Odense University hospital, Denmark

## Abstract

Cystic fibrosis (CF) is the most common genetic disease among Caucasians, and accordingly the cystic fibrosis transmembrane conductance regulator (CFTR) protein has perhaps the best characterized disease mutation spectrum with more than 1,500 causative mutations having been identified. In this study, we took advantage of that wealth of mutational information in an effort to relate site-specific evolutionary parameters with the propensity and severity of CFTR disease-causing mutations. To do this, we devised a scoring scheme for known CFTR disease-causing mutations based on the Grantham amino acid chemical difference matrix. CFTR site-specific evolutionary constraint values were then computed for seven different evolutionary metrics across a range of increasing evolutionary depths. The CFTR mutational scores and the various site-specific evolutionary constraint values were compared in order to evaluate which evolutionary measures best reflect the disease-causing mutation spectrum. Site-specific evolutionary constraint values from the widely used comparative method PolyPhen2 show the best correlation with the CFTR mutation score spectrum, whereas more straightforward conservation based measures (ConSurf and ScoreCons) show the greatest ability to predict individual CFTR disease-causing mutations. While far greater than could be expected by chance alone, the fraction of the variability in mutation scores explained by the PolyPhen2 metric (3.6%), along with the best set of paired sensitivity (58%) and specificity (60%) values for the prediction of disease-causing residues, were marginal. These data indicate that evolutionary constraint levels are informative but far from determinant with respect to disease-causing mutations in CFTR. Nevertheless, this work shows that, when combined with additional lines of evidence, information on site-specific evolutionary conservation can and should be used to guide site-directed mutagenesis experiments by more narrowly defining the set of target residues, resulting in a potential savings of both time and money.

## Introduction

Cystic fibrosis (CF) is a recessive genetic disease that is caused by mutations to the gene encoding the cystic fibrosis transmembrane conductance regulator (CFTR) protein [1]. The CFTR protein serves as a channel that regulates the transport of chloride and sodium ions across cell membranes in epithelial tissues. Abnormal ion transport caused by defective (mutated) CFTR channels leads to the accumulation of viscous mucus, particularly in the lungs, resulting in difficulties in breathing, poor growth, airway and sinus infections, and infertility, among other symptoms.

CF is the most common hereditary disease among Caucasians, with ∼1 out of 29 individuals of European descent carriers of a CF causing allele [2]. Owing to the severity of this genetic disease, and to its frequency of occurrence, the CFTR gene has been the subject of an intense research focus for years. These efforts have led to the accumulation of a large collection of >1,500 identified disease-causing mutations in the CFTR gene, which is stored and disseminated by the Cystic Fibrosis Mutation Database (http://www.genet.sickkids.on.ca/). As such, CFTR has one of the best characterized spectra of disease-causing mutations for any human gene.

For this study, we were interested in relating the mutation spectrum of CFTR to its site-specific evolutionary rate profile. The rationale underlying this investigation was the notion that evolutionary information can be used to guide inferences about function. For example, in the case of CFTR or other disease related genes, one may be able to employ evolutionary information to predict which residues are most likely to be mutated in cases of disease. In fact, evolutionary inferences are already widely employed to inform studies of structure and function in this way [3], [4], [5], [6], [7].

The most straightforward approach to this problem rests on the assumption that functionally important residues will be subject to greater selective constraint and thus less likely to change over time [8]. According to this rationale, disease-causing mutations are most likely to correspond to evolutionarily conserved positions in a gene (protein) sequence due to their functional importance. This assumption has been confirmed in numerous comparative studies of human disease genes [9], [10], [11], [12]. Nevertheless, there are conflicting results suggesting that disease genes can evolve under less selective constraint than non-disease genes [13]. This may be attributable to the fact that disease genes actually evolve under moderate selective constraint based on phenotypes that are intermediate in severity between those caused by lethal mutations and those caused by mutations with little or no effect [13]. Consistent with this idea, some sets of disease-causing mutations have been shown to map to sites that are moderately, as opposed to fully, conserved over evolutionary time [14]. Even more striking is the fact that mutated residues that are disease-causing in human have been shown to represent the wild type state in the same proteins from related species because of compensatory changes at other sites in the same protein [15], [16]. Considered together, these conflicting results suggest that the precise nature of the relationship between evolutionary rates and disease-causing mutations is currently unclear.

In this study, we addressed two specific questions with respect to this relationship as it pertains to the CFTR gene: 1) what measure of site-specific evolution best reflects the CFTR mutation spectrum, and 2) how much sequence variation should be included to maximize the correlation between site-specific evolution and mutation in CFTR. In addition to providing information on the relationship between mutation and evolution in CFTR, we hope that answers to these questions can also help investigators make better use of evolutionary information in their own experimental design and interpretation.

## Results and Discussion

### Mutation Spectrum of CFTR

**Figure 1:**
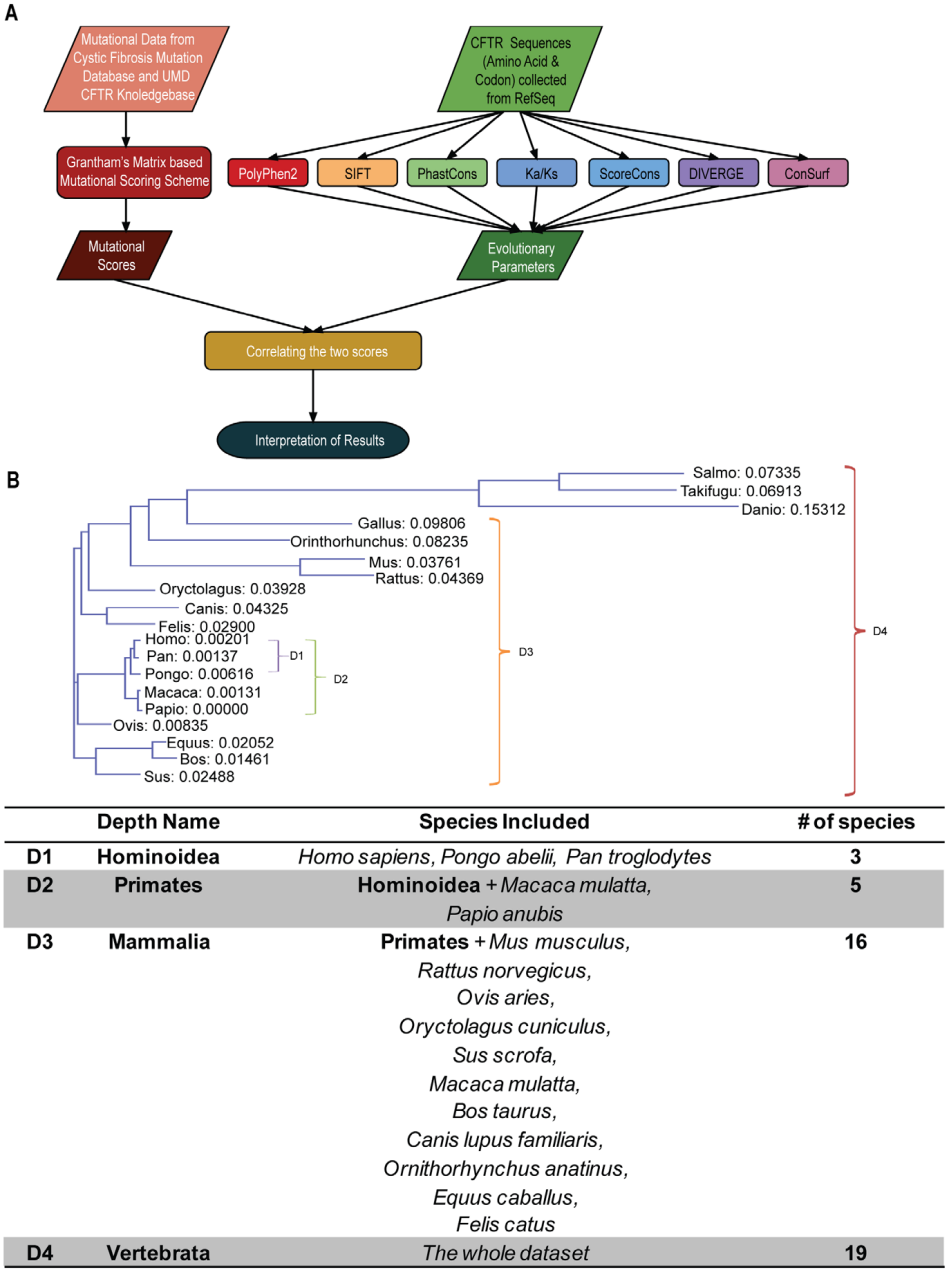
Scheme of the analysis used in this study. (A) Flow chart illustrating the joint analysis of CFTR mutation data from the Cystic Fibrosis Mutation Database and site-specific evolutionary metrics based on seven different comparative methods. (B) CFTR phylogenetic tree and associated list of species analyzed indicating the four ascending evolutionary depths used in the study.

In order to define the mutation spectrum of the CFTR gene, previously characterized mutations were taken from the Cystic Fibrosis Mutation Database (http://www.genet.sickkids.on.ca/) (Figure 1A). The vast majority of these mutations are thought to be disease-causing and are primarily related to cystic fibrosis along with a minority of mutations that cause congenital absence of the vas deferens (CBAVD) but no known pulmonary defect as would be associated with classic CF. Our analysis of disease related CFTR mutations was limited to exonic sequences and non-synonymous mutations in order to facilitate comparisons with evolutionary parameters, which are computed using cross-species comparisons. Intronic sequences are not sufficiently conserved over time to allow for accurate comparisons between more distantly related vertebrate species, and only information on non-synonymous coding sequence mutations, which change the encoded amino acid sequence, can be compared with evolutionary methods that utilize protein sequence comparisons. As of December 2011, a total of 1,404 individual CFTR exonic mutations, covering 670 out of the 1,480 CFTR codons, were available for analysis.

**Figure 2:**
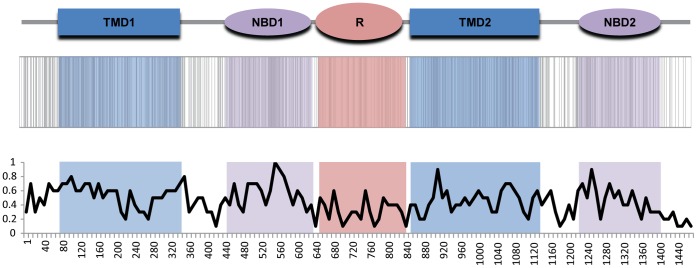
Locations of disease-causing mutations along the CFTR protein sequence. The domain architecture of CFTR is shown with TMD-transmembrane domain, NBD-nucleotide binding domain and R-regulatory domain. The locations of protein residues that are known to be mutated in CF disease cases are indicated with gray vertical bars below the domain architecture, and the average numbers of mutated residues are shown for 10-residue long sliding windows along the length of the protein.

**Figure 3:**
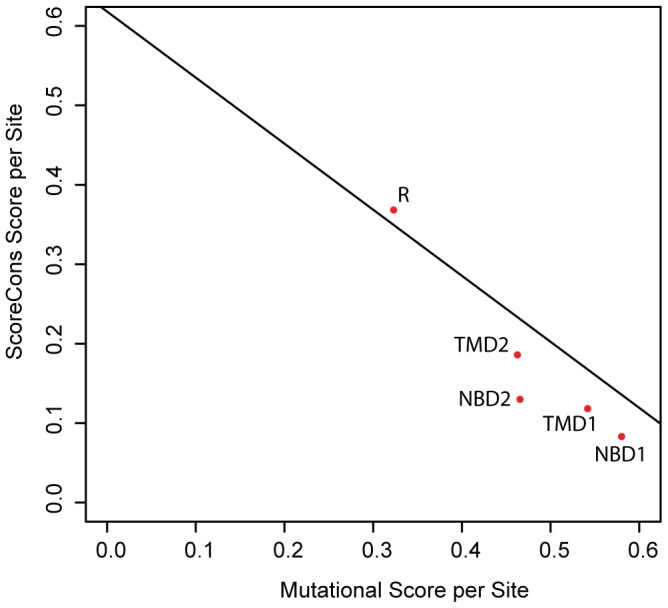
Correlation between evolutionary and mutational scores for individual CFTR domains. The average ScoreCons per-site score for each of the five CFTR domains was regressed against the average mutational per-site score for the domains.

CFTR exonic disease-causing mutations are broadly distributed across the protein coding sequence and map to the coding regions for all five of the known CFTR protein domains: the two transmembrane domains (TMDs), the two nucleotide binding domains (NBDs) and the regulatory (R) domain (Figure 2). Nevertheless, there are significant differences in the average number of mutations per site for the domains. The 5′ NBD1 domain coding sequence has the highest number of mutations per site, and the R domain region has the fewest mutations per site. A similar bias of disease-causing mutations in the NBD1 region has previously been observed [17]. This may reflect differences in the functional relevance of the domains as well as their structural constraints. The NBD domains encode enzymatic activity, the hydrolysis of ATP, and are highly structured, whereas the R domain is non-enzymatic and unstructured [18], [19]. Thus, mutations to the NBD1 coding region may be more prone to cause disease than mutations to the R domain region. There may also be an evolutionary dimension to this observation, and indeed features of structure/function and evolution are clearly not mutually exclusive. The NBDs are conserved between CFTR proteins and are found in related ABC transporters, whereas the R domain is specific to CFTR and not found in other ABC transporters. Again, this may point to a lower tolerance for mutations in NBD coding regions and the corresponding excess of observed disease-causing mutations in the NBD1 region. Consistent with this idea, there is a strong positive correlation between the prevalence of disease-causing mutations among the CFTR domains and their levels of evolutionary constraint (Figure 3). The relationship between the CFTR mutation spectrum and the evolution of the gene will be explored in greater depth in the rest of the manuscript. There also appears to be a bias for mutations in the 5′ end of the CFTR gene with both TMD1 and NBD1 showing an excess of disease-causing mutations per site relative to all three downstream domains, although the reasons for this excess of 5′ mutations are less clear.

**Figure 4:**
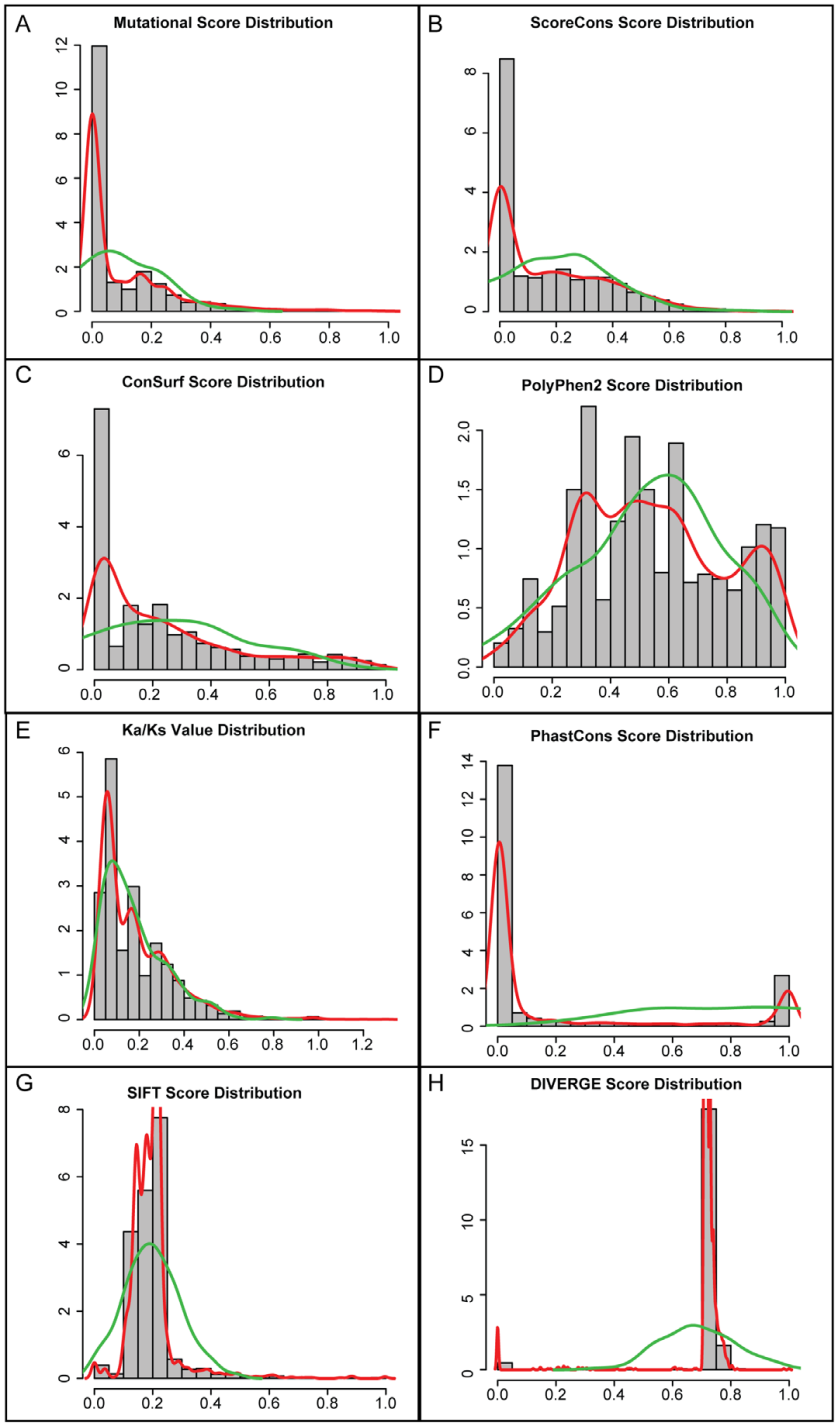
Probability distributions of the CFTR per-site mutational and evolutionary scores. For the mutational score (**A**) and each of the seven evolutionary scores (**B**–**H**), observed distributions are shown in gray (20 bins) and red (smoothed distributions). The best fitting theoretical distributions are shown in green.

A scoring scheme for the CFTR exonic disease-causing mutations analyzed here was implemented to allow for direct comparisons between the CFTR mutation spectrum and CFTR site-specific evolutionary parameters (Figure 1A). Scores were calculated in units of individual codons and are based on the amino acid changes entailed by the observed disease-causing mutations. These amino acid changes are scored using the Grantham chemical difference matrix, which measures the mean chemical distance between residues for three different properties [20]. The Grantham matrix was chosen for the CFTR mutation scoring scheme based on previous observations showing that this measure is significantly correlated with the clinical likelihood of a number of different human diseases [21]. Scores were scaled from 0 to 1 under this scheme; relatively greater deviations from the wild-type CFTR sequence are given a higher score, and codons with no mutations are given a score of 0. Details of this CFTR mutation spectrum scoring scheme can be found in the Materials and Methods section. The distribution of CFTR mutation scores is peaked at zero, *i.e.* codons with no mutations in the Cystic Fibrosis Mutation Database, and has a long tail of non-zero scores that are approximately normally distributed (Figure 4A).

### Site-specific Evolution of CFTR

The goal of this study was to relate the CFTR mutation spectrum to the relative evolutionary constraints on the CFTR sequence. In order to do this, seven different measures of site-specific evolutionary constraint were computed across four depths of evolutionary relatedness for CFTR sequences that are found among vertebrates. Descriptions of each of these evolutionary parameters, along with conceptual details for how they are calculated, are provided in Table 1. The seven evolutionary parameters can be conceptually partitioned into three groups: conservation, probabilistic, and selection (Table 1). ScoreCons and ConSurf are measures of site-specific evolutionary conservation based on amino acid sequence comparisons; the Polyphen2, SIFT, DIVERGE and PhastCons measures form a heterogeneous group with methods that measure site-specific evolution, or relative effects of site-specific changes, based on probabilistic analyses of multiple sequence alignments, and the ratio of non-synonymous (*Ka*) to synonymous (*Ks*) substitution rates (*Ka/Ks*) is a nucleotide sequence (codon)-based measure of selective constraint. It should be noted that since this study compared quantitative levels of site-specific sequence constraint produced by different evolutionary methods, we were not able to include methods that produce qualitative data as output, such as the DETECTER program [4] that yields lists of potentially tolerated versus intolerated residues at each position.

**Table 1:** Site-specific measures of evolutionary constraint.

Parameter	Description	Class
**(1) ScoreCons**	ScoreCons provides a measure of site-specific evolutionary conservation [30]. The highest score represents themost conserved position in a protein. The scores provided account for amino acid frequency, utilizes thestereochemical properties of the amino acids within the substitution matrix and normalizes against theredundancy in the alignment [31]. ScoreCons uses a type of sum-of-pairs scoring with scores ranging from 0 to 1.	Conservation
**(2) ConSurf**	Given an MSA and the corresponding phylogeny, ConSurf calculates position-specific conservation scores usingthe empirical Bayesian or Maximum Likelihood algorithms [32], [33]. The scores are normalized, so that the averagescore for all residues is 0, and the standard deviation is 1. The conservation scores calculated by ConSurf area relative measure of evolutionary conservation at each sequence site of the target chain. The lowest scorerepresents the most conserved position in a protein. It does not necessarily indicate 100% conservation (*e.g.* nomutations at all), but rather indicates that this position is the most conserved in this specific protein calculatedusing a specific MSA.	Conservation
**(3) PhastCons**	PhastCons [34] identifies conserved sites based on a statistical model of sequence evolution calleda phylogenetic hidden Markov model. PhastCons considers *n* species and their phylogeny. It then usesstatistical models of nucleotide substitution that allow for multiple substitutions per site and for unequalrates of substitution between different pairs of bases. Its output is a probability that each nucleotidebelongs to a conserved element.	Probabilistic
**(4) DIVERGE**	DIVERGE [35] implements a two-step statistical testing for site-specific rate shifts along the input usertree and thus predicts candidate amino acid residues responsible for functional divergence based on posterioranalysis. Its output is a set of posterior probabilities for specific sites (possibly those responsible forthe functional divergence).	Probabilistic
**(5) PolyPhen2**	The Polyphen algorithm [3], [6] takes as input the query protein sequence and the SNP with its positionwhich is to be analyzed. Based on an internal pipeline, it then proceeds to calculate the probability that the inputSNP is deleterious for the sequence. The pipeline first performs a BLAST search to retrieve high-scoringsequence pairs by identity. Using these, it constructs an initial MSA which is refined in the subsequent steps byperforming clustering. From this refined MSA, the algorithm scores a series predefined amino acid residue andstructural characteristics for the input positions and jointly considers these features to calculate theNaïve Bayes probability that this mutation is damaging.	Probabilistic
**(6) SIFT**	SIFT [5] takes into consideration that changes at well-considered positions tend to be deleterious in nature.SIFT uses a two-step algorithm in which the first step involves the identification of closely relatedsequences and construction of an MSA from these sequences. Once an MSA is obtained, the algorithm calculatesthe probability that the given site belongs to the conserved sites of the sequence.	Probabilistic
**(7) Ka/Ks**	Estimation of selective pressures acting at the level DNA coding sequences (CDS) can be determined byetermined by estimating the ratio of non-synonymous (*Ka*) to synonymous (*Ks*) rates of CDS substitution.An estimate of *Ka* that is significantly lower than *Ks* provides evidence for the action of purifying selection(*i.e.* evolutionary constraint). To estimate the selective constraint at each codon, the ratio (ω) of *Ka/Ks* wascalculated using the Selecton server [36].	Selection

Conceptual summaries for each of the seven algorithms used to calculate site-specific evolutionary constraint are provided, and the algorithms are classified according to the general class of approach that they use.

Beyond the simple classification scheme for the methods described above, each of these seven approaches for measuring site-specific evolutionary rates can be considered to have their own strengths and weaknesses prior to comparative analysis. For instance, the conservation based method ScoreCons has the advantage of being conceptually straightforward, but may be considered to be overly simplistic since it does not take information on the phylogenetic relationships between sequences into account as do the ConSurf and PhastCons methods. PhastCons also has the advantage of allowing for multiple substitutions per site and unequal rates of substitution across lineages, whereas the fact that it was designed for calculating nucleotide diversity may place it at a disadvantage here compared to methods that are designed for amino acid sequence comparison. *Ka/Ks* is also a nucleotide ba[sed measure and suffers an even greater disadvantage of saturation of *Ks*, owing to the rapid accumulation of multiple substitutions at synonymous sites, for divergent sequence pairs. Polyphen2 stands out from all of the methods employed here in that it uses its own custom sequence search and alignment pipeline and takes into account a number of different aspects of sequence conservation and structural environment when computing its score. While the PolyPhen2 alignment protocol provides for quality control, the quality of the alignments produced can not be manually verified and/or refined by the user. We provide additional detail on the discrepancies between the methods along with their relative strengths and weaknesses in Table S1.

As with the mutation scoring scheme, the scores for six of these measures of site-specific evolution were scaled to range from 0 to 1, with 0 representing the highest conservation and 1 representing the maximum variability. *Ka/Ks* was not scaled in this way since it naturally takes on values over this range but is unbounded, with a sparse tail, above the value of 1. Distributions for each of these site-specific evolutionary scores, at depth 4 with all vertebrate sequences included, are shown in Figure 4 panels B–H. The ScoreCons (Figure 4B) and ConsSurf (Figure 4C) distributions most closely resemble the distribution of CFTR mutation scores (Figure 4A) with a high peak at 0 and a long tail of non-zero scores that are approximately normally distributed. The Polyphen2 scores (Figure 4D), while also approximately normal, show by far the broadest and most uniform distribution across the score range. *Ka/Ks* shows the only distribution (Figure 4E) that is best approximated by a gamma function, and most values are found closer to 0 (*i.e*. maximum selective constraint). The PhastCons distribution (Figure 4F) is extremely bimodal with the highest peak at 0 (no change) and a smaller peak at 1 (maximum change). SIFT shows a peaked normal distribution (Figure 4G) with most values close to the more conserved end of the spectrum, whereas the DIVERGE distribution (Figure 4H) is bimodal with a peak closer to the higher end of relative variation.

**Figure 5:**
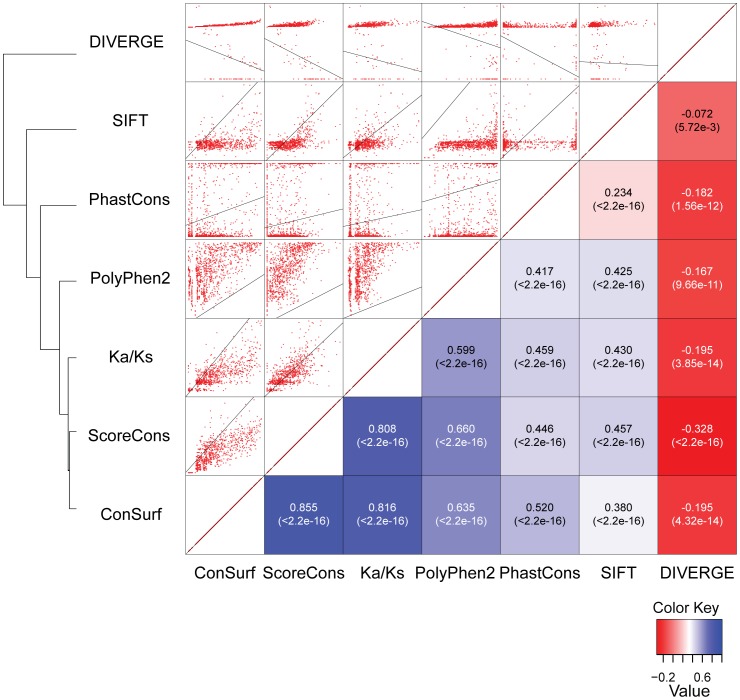
Pairwise correlations between per-site scores and relationships for the seven evolutionary metrics. Individual per-site CFTR scores were regressed for all pairs of methods. Scatter plots are shown about the diagonal and Pearson correlation coefficients (PCC), along with their associated *P*-values, shown below the diagonal. The evolutionary metrics are related using hierarchical clustering of the PCC values.

The relationships among these score distributions were evaluated by performing pairwise correlations between the CFTR site-specific variation profiles that they generated followed hierarchical clustering on the resulting correlation coefficients. Pearson correlation was used to do this in light of the approximately normal distributions of the scaled and transformed measures evaluated here (Figure 4). The first observation from this analysis is that the seven measures of site-specific variation evaluated here can yield substantially distinct site-specific variation profiles (Figure 5). This is despite the fact that the measures are being applied to the same CFTR multiple sequence alignment, with the exception of Polyphen2 which creates its own alignment. The Pearson correlation coefficients (PCCs) range from −0.33 to 0.86, and ∼30% of the PCC values (6 out of 21) are negative. DIVERGE in particular appears to be an outlier since it is significantly negatively correlated with 5 out of 6 of the other measures that were evaluated. The variability observed for these pairwise correlations suggests that the different site-specific variation measures employed here capture different aspects of the evolutionary process.

The second observation from this analysis is that the different measures compared tend to group according to the class of algorithm that they represent (Figure 5). The two conservation measures (ConSurf and ScoreCons) are grouped most closely, followed by Ka/Ks which also measures selective constraint and then the probabilistic methods follow. Polyphen2 and PhastCons occupy the most intermediate positions on the PCC dendogram.

### Relating CFTR Mutation and Evolution

**Figure 6:**
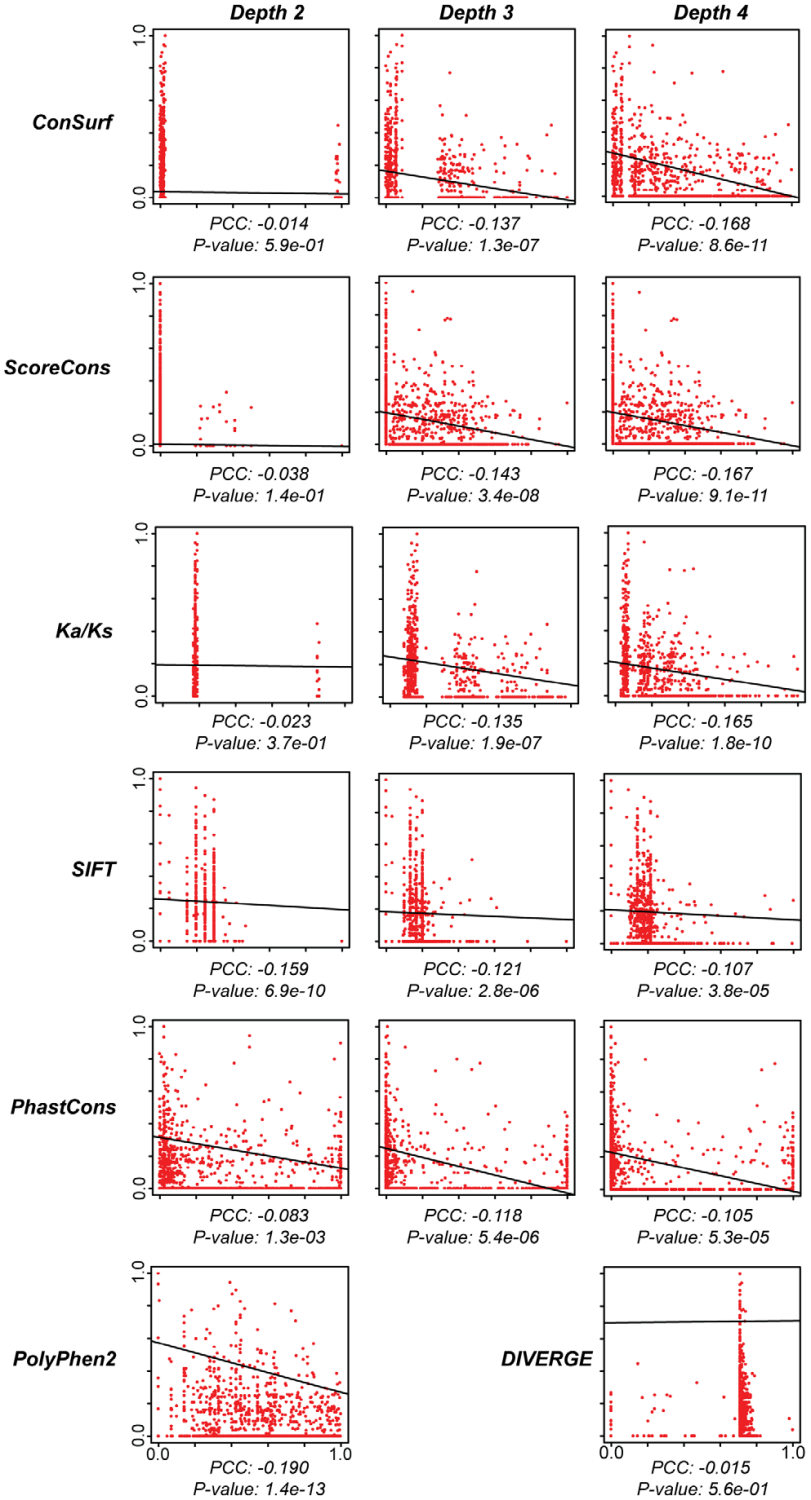
Pairwise correlations between CFTR mutational scores and scores from seven evolutionary metrics. Mutational scores were regressed against the various evolutionary scores and the resulting Pearson correlation coefficients (PCC) and P-values are shown. The results for all evolutionary metrics, except for PolyPhen2 and DIVERGE, are shown for evolutionary depths 2–4. PolyPhen2 employs an intrinsic similarity search to achieve maximum evolutionary depth, and DIVERGE could only be run at depth 4 (see Table 2).

Having established the variability of the different site-specific evolution measures used here, we then related the CFTR mutation spectrum to the evolutionary site-specific variation profiles generated by these different measures (Figure 6 and Table 2). This was done across a series of increasing evolutionary depths (1–4), each of which contains more distantly related species (Figure 1). The shallowest evolutionary depth 1, which only contained Hominoidea, did not provide enough sequence variation to yield any information for the comparison with the CFTR mutation spectrum. Accordingly, only results from depths 2–4 are presented here. The program DIVERGE measures differences between two user defined clades, and therefore was only used at the maximum depth 4 to ensure adequate sequence representation in each clade. Polyphen2 is the only program used here that conducts its own sequence search and computes its own multiple sequence alignment. The variation present in the resulting Polyphen2 alignment is roughly equivalent to depth 4 since it captures a diverse set of CFTR sequences using an internal BLAST similarity search.

**Table 2:** Pairwise correlations between CFTR mutation scores and site-specific evolutionary constraint values.

		Depth 2: Primates (5)		Depth 3: Mammalia (16)		Depth 4: Vertebrata (19)	
	Parameter	PCC	*P*-value	PCC	*P*-value	PCC	*P*-value
**1**	**PolyPhen2**	Not Applicable^2^		Not Applicable^2^		−0.191	1.44e-13
**2**	**ConSurf**	−0.014	6.00e-01	−0.137	1.31e-07	−0.168	8.56e-11
**3**	**ScoreCons**	0.038	1.50e-01	0.143	3.40e-08	−0.167	9.11e-11
**4**	**KaKs**	−0.0232	3.70e-01	−0.135	1.99e-07	−0.165	1.77e-10
**5**	**SIFT**	−0.159	6.89e-10	−0.121	2.82e-06	−0.107	3.82e-05
**6**	**PhastCons**	−0.083	1.31e-03	−0.118	5.44e-06	−0.105	5.33e-05
**7**	**DIVERGE**	No Results^1^		No Results^1^		0.015	5.62e-01

The CFTR mutational score spectrum was regressed against site-specific evolutionary constraint values for seven different evolutionary metrics across a range of increasing evolutionary depths (see Figure 6). For each comparison, the Pearson correlation coefficient (PCC) and associated *p*-value are shown. Evolutionary methods are ranked according to the best correlations.

1 DIVERGE produced all 0 posterior probabilities due to insufficient number of sequences.

2 PolyPhen2 requires only single amino acid sequence to do the scoring and hence the depth-level concept nullifies in this case.

In general, CFTR mutation scores are negatively correlated with measures of site-specific evolutionary variation. In other words, CFTR sites that are more conserved are more likely to cause disease when mutated. This observation is both intuitive and expected, since functionally important sites are more likely to be conserved than less important sites. Nevertheless, there are substantial differences in the results across different evolutionary depths as well differences in the efficacies of the various evolutionary measures in terms of correlating with the CFTR mutation spectrum. Overall, depth 4 yields the best results (*i.e.* the strongest correlations with the CFTR mutation spectrum). However, this is not always the case; SIFT shows the highest negative correlation with the least overall variation at depth 2.

Use of the program PolyPhen2 provides the strongest correlation with the CFTR mutation spectrum (Figure 6 and Table 2). This result is consistent with previous comparisons showing that PolyPhen2 was the best program for predicting the deleterious effects of non-synonymous protein coding sequence mutations [3]. In fact, this result may not be surprising considering the fact that the quantitative output from PolyPhen2 is distinct from the other measures of site-specific evolutionary rates compared here in the sense that it is actually a probability that any particular mutation is damaging to the function of the protein, and in this way may represent a more directly relevant comparison to disease causing mutations than the other measures. The superior performance of the Polyphen2 algorithm observed here may also be attributed to the fact that Polyphen2 incorporates additional functionally relevant information beyond what can simply be found in a multiple sequence alignment. For example, Polyphen2 incorporates structural information vis-à-vis the results of sequence comparisons it does against the PDB structural database [22]. It also uses protein domain information from Pfam [23] as well as information on the nature of the observed nucleotide substitutions.

The next best correlations are obtained when the conservation measures ConSurf and ScoreCons are used at depth 4, and then *Ka/Ks* at depth 4 provides a similar level of correlation to ConSurf and ScoreCons. While PolyPhen2 provides ∼12% better performance, in terms of correlating with the mutation spectrum, the differences between the correlation values provided by PolyPhen2 and the conservation measures are not statistically significant. This suggests that the conservation *per se* provides the most reliable signal for predicting the effects of mutations in the CFTR gene. In addition, the more available information there is, in terms of sequence divergence, the better the correlation with mutation will be.

### Effect of Alignment Algorithm Choice and Sequence Selection

The results reported above are based on the use of a single multiple alignment algorithm (CLUSTALW) and a relatively limited set of representative CFTR sequences. We further evaluated how the choice of the alignment algorithm affects the quality of the alignment along with the relationship between CFTR mutation and evolution by comparing results obtained using four different programs, CLUSTALW [24], ProbCons [25], MUSCLE [26], [27], T-Coffee [28]. These programs were chosen because they are widely used, considered to be reliable and each employs a fundamentally different alignment algorithm. Furthermore, the T-Coffee alignment method was run using the T-Coffee algorithm alone and in a combined mode that merges results from multiple programs in an effort to yield a more reliable alignment.

The alignment methods were compared by taking the average site-specific conservation scores as a quantitative measure for alignment quality (see http://tcoffee.crg.cat/apps/tcoffee/do:core). This was first done on the same set of 19 NCBI RefSeq CFTR amino acid sequences described above for evolutionary depth 4 (Figure 1). The results of this analysis suggest that CLUSTALW yields the most reliable multiple sequence alignment (Figure S1). This result was somewhat unexpected since CLUSTALW is the oldest of these methods and sometimes considered to be obsolete. This result may simply reflect the fact that the CFTR sequences analyzed here are highly conserved and thus do not necessitate the use of a more advanced sequence alignment algorithm, such as T-Coffee which has been shown to perform better with more distantly related sequences. It may also reflect the fact that the alignments evaluated here consist of relatively few sequences; MUSCLE for instance has been shown to perform well on large sequence sets. The effects of including additional sequences are considered below. In any case, the differences in the alignment qualities produced by the different methods, while statistically significant, are somewhat marginal.

Having evaluated the relative performance of the alignment algorithms in this way, we also checked to see if the different alignments produced changed the nature of the relationship between CFTR mutation and evolution. To do this, we recomputed the correlations between site-specific evolutionary constraint measures and mutation scores across the different alignments where possible. This comparison could not be made for programs that use their own alignment methods, *i.e.* PolyPhen2, PhastCons and *Ka/Ks*. The relative ranks of the site-specific evolutionary constraint methods did not change appreciably when different alignment methods were used (Table S2). The position of ConSurf changes slightly with respect to the ScoreCons and *Ka/Ks* methods, but this was a relatively minor change since these methods were basically indistinguishable using CLUSTALW. These findings are consistent with the marginal differences in alignment quality produced by the different methods (Supporting Information Figure S1). Nevertheless, in cases where sequences are more diverged and alignment qualities differ more substantially than seen here, users may be cautioned that the results could change more dramatically.

We also evaluated the effect of including additional sequences in the analysis. We initially used only 19 CFTR sequences from the NCBI RefSeq database because these sequences are highly curated, and thus reliable, and also make up a representative set that evenly spans the phylogenetic diversity at evolutionary depth 4 (Figure 1). However, there are numerous additional CFTR sequences available including many closely related sequences along with sequences that result from automated gene model predictions and thus are likely to be less reliable. We included all such available sequences together with the 19 NCBI RefSeq sequences in an expanded set of 192 CFTR sequences, and compared the performance of this set with respect to both alignment quality and the correlation between CFTR evolution and mutation. The expanded set results in the lowest quality alignment observed (Figure S1) and generally lower correlations between CFTR site-specific evolutionary rates and mutation scores (Table S3). Nevertheless, the overall relative performance of the site-specific evolutionary rate measures did not change appreciably. Experimentalists should be cautioned again that the robustness of these results to sequence sets of different sizes may be attributable to the fact that CFTR represents a highly conserved set of orthologous sequences with a relatively narrow evolutionary range.

### Predicting CFTR Mutations with Evolutionary Data

While the correlations between the CFTR mutation spectrum and the site-specific evolutionary rates are highly statistically significant, owing to the large number of CFTR sites analyzed (*n* = 1,480), the PCC values are modest at best. The highest performing evolutionary measure, PolyPhen2, yields a PCC of −0.19, which corresponds to an *r^2^* value of 0.036. In other words, only 3.6% of the variance in the CFTR mutation score can be predicted by the variance in PolyPhen2 site-specific evolution scores. Based on this observation, one must exercise caution when trying to use evolutionary information to specifically predict which sites in a gene (protein), such as CFTR, are most likely to be mutated in disease cases.

**Figure 7:**
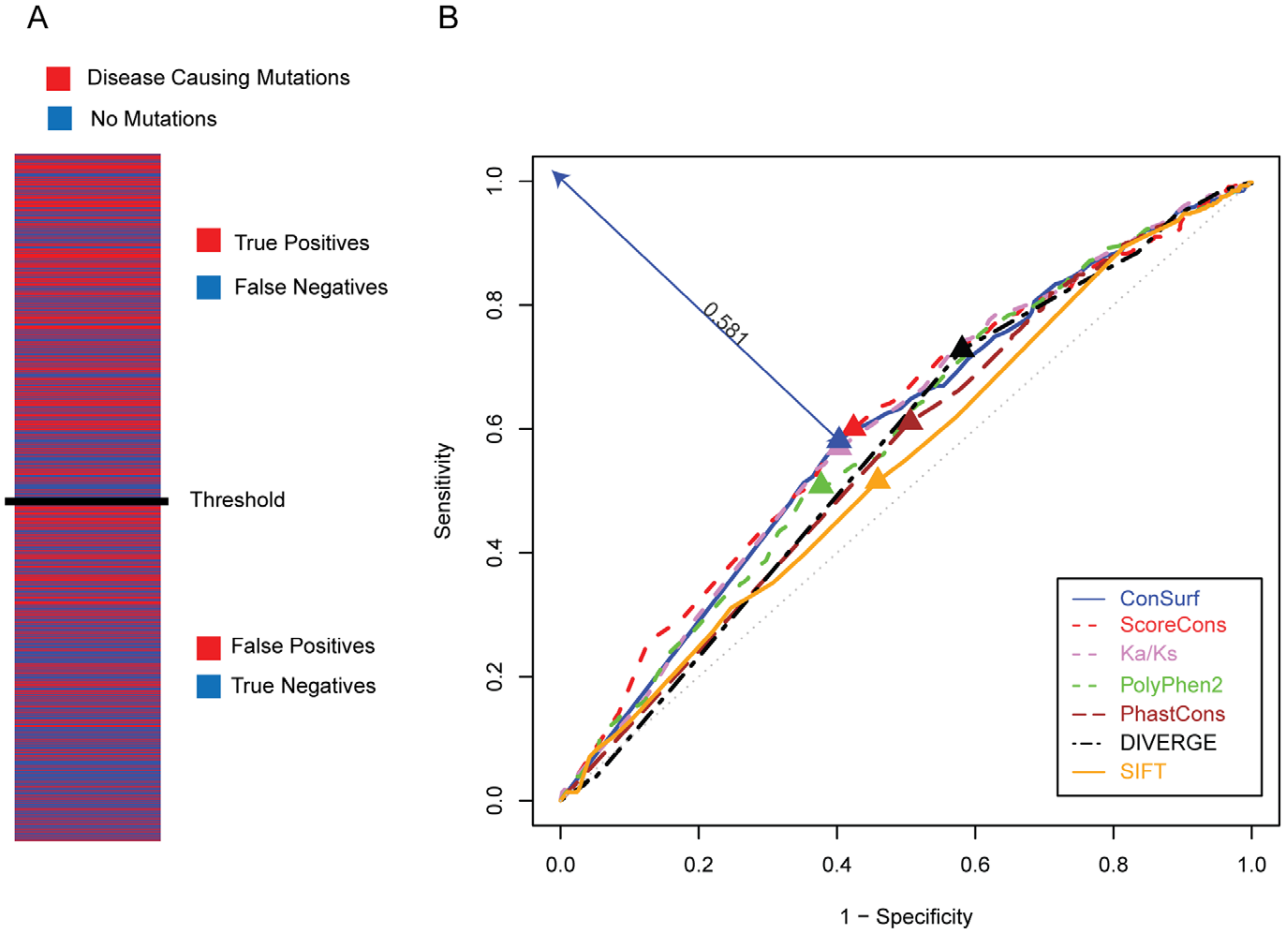
Predictive power for the seven evolutionary metric scores. (A) Scheme of the prediction power analysis. Residues mutated in CFTR disease cases are shown in red and non-mutated residues are shown in blue. Residues are ranked in descending order according to an evolutionary conservation metric. A conservation score threshold is chosen; residues above this threshold are predicted to be mutated and those below are predicted to be non-mutated. This allows for the classification of each residue as a true positive, false negative, false positive or true negative according to its classification and its location above or below the score threshold. (B) Receiver operating curve (ROC) analysis was used to evaluate the predictive power of the seven evolutionary metric scores and to maximize the trade-off between sensitivity and specificity. For each evolutionary metric, the point along the ROC curve that minimizes the Euclidean distance between the coordinates y = observed sensitivity, x = observed 1-specificity and the perfect predictor coordinate of y = 1, x = 0 is taken as the optimal threshold (indicated with triangles). An example of the minimal Euclidean distance for the ConSurf method is shown. For the thresholds chosen in that way, sensitivity and specificity are averaged to come up with a ranked predictor value for each evolutionary metric.

To explore this idea further, we attempted to use the site-specific evolution measures to predict sites that have disease-causing mutations in CFTR. To do this, CFTR sites were classified with a simple binary designation as to whether they contain a disease-causing mutation or not (Figure 7A). Then each site-specific evolutionary measure was used across a series of thresholds above (or below) which all sites were predicted as mutated (or not). This approach allowed for true positives (TP) to be counted as the number of observed mutated sites above the evolutionary score threshold, and false positives (FP) were counted as the number of observed mutated sites below the score threshold. Similarly, false negatives (FN) were counted as the number of sites above the threshold not observed to be mutated, and true negatives (TN) were the number of sites below the threshold not observed to be mutated. Receiver operating characteristic (ROC) curves were then plotted to evaluate the trade-off between sensitivity [(TP/(TP+FN)] and specificity [TN/(FP+TN)] in predicting mutated sites over different threshold values (Figure 7B). The ROC curves were also used to identify the site-specific evolution rate threshold values for each measure that maximized this trade-off by computing the minimum Euclidean distance between the ROC curves and the upper left corner of the plots, which represents a theoretically perfect predictor (*i.e.* maximum sensitivity and specificity).

In contrast to the results of the correlation analysis, the conservation based measures ConSurf, ScoreCons and Ka/Ks were both extremely close to one-another based on the ROC curve analysis and also scored the highest with respect to the trade-off between sensitivity and specificity (Figure 7B and Table 3). In other words, these methods yielded the best performance in terms of the prediction of individual disease-causing mutation sites (Figure 7 and Table 3). However, each of these methods achieved harmonic means of sensitivity and specificity below 60%. These results indicate that even the most reliable evolutionary metrics only perform marginally well in predicting the disease-causing potential of individual CFTR mutations. Nevertheless, use of this information, together with additional functional and/or structural considerations, can help guide mutagenesis experimental approaches.

### Recommendations for Experimentalists

This study was conceived and executed in an attempt to assess the extent to which evolutionary information can inform functional studies of human disease genes (proteins). To do this, we sought to evaluate whether the CFTR disease-causing mutation spectrum was related to site-specific measures of evolutionary constraint, and if so, to determine which evolutionary metrics yielded the best correlations with, or predictions of, CFTR disease mutations. The hope was that answers to those questions could provide guidance to experimentalists in terms of how to best select individual residues, or sets of residues, for functional interrogation.

The widely used program PolyPhen2 yields the best correlation values between the CFTR mutation spectrum and site-specific evolutionary constraint values. This may reflect the fact that PolyPhen2 uses a wide variety of information sources, when available, and has been continuously refined over several years. Nevertheless, the differences between the correlation values yielded by PolyPhen2 versus those of the more straightforward conservation measures ConSurf and ScoreCons are not statistically significant. This suggests that conservation itself is the most important feature to be considered when attempting to measure the functional impact of individual mutations. Another relevant take home message from this study is that it is important to include as much variation as possible, while controlling for alignment quality, from any individual protein family when attempting to use evolutionary inferences to inform function.

Consistent with the notion that conservation *per se* is the most important aspect of evolution as it relates to function, when the evolutionary metrics were compared with respect to their ability to predict individual CFTR sites as disease-causing mutations or non-mutated in disease, the straight conservation measures performed the best. These differences in relative performance may reflect the fact that the correlations reported rely on a continuous mutation score for all CFTR residues, whereas the disease-causing mutation prediction is a binary classification that distinguishes sites as being implicated in CF disease or not. In either case, the fraction of variability in mutation scores explained by variation in PolyPhen2 scores is low (3.6%), and the sensitivity (58%) and specificity (60%) values for the prediction of disease-causing residues by ConSurf were marginal. These data point to the fact that site-specific evolution constraint levels are not deterministic with respect to the functional role of any particular residue or position. They may also reflect, to some extent, the incompleteness of the CFTR disease-causing mutation spectrum. Thus, experimentalists must use caution when employing site-specific evolutionary conservation measures to inform functional studies. Nevertheless, the signal of the relationships between evolution and mutation observed here is quite strong and far greater than could be expected by chance alone. Thus, judicious use of such evolutionary information, together with additional lines of evidence, should prove to be quite useful in narrowing down sets of residues to be experimentally studied. A reduction in the space of possible residues to be investigated can in turn result in substantial savings for experimentalists in terms of time, effort, and money.

**Table 3:** Predictive value of the seven evolutionary metrics for individual disease-causing CFTR mutations.

Evolutionary Constraint	Sensitivity	Specificity	Harmonic Mean	Euclidean Distance from Perfect Predictor
**1 ConSurf**	0.581	0.597	0.589	0.581
**2 ScoreCons**	0.601	0.576	0.588	0.582
**3 Ka/Ks**	0.57	0.597	0.583	0.590
**4 PolyPhen2**	0.508	0.623	0.560	0.620
**5 PhastCons**	0.611	0.494	0.547	0.638
**6 DIVERGE**	0.728	0.419	0.532	0.641
**7 SIFT**	0.516	0.541	0.528	0.667

For each evolutionary metric, ROC analysis was used to evaluate the trade-off between sensitivity and specificity over a range of score thresholds, and the optimal threshold was taken as position of on the curve that minimizes the Euclidean distance from the perfect predictor (see Figure 7 and text). For these thresholds, evolutionary metrics are ranked according to the harmonic mean between the sensitivity and specificity of predicting individual CFTR residues as disease-causing or non-mutated.

We conclude here by providing a series of concrete recommendations for experimentalists for how site-specific mutation studies can be set up to take account of evolutionary information based on our observations in this study. There are four specific concept areas that experimentalists should consider when doing so: 1) sequence database source and selection, 2) evolutionary breadth and depth of represented sequences, 3) alignment programs and quality measures, and 4) use of and consensus among several methods of site-specific evolutionary constraint. Sequence databases are highly redundant and include numerous gene model predictions and low quality sequences, for instance sequences from low coverage genome projects, which should be avoided when possible. The easiest way to do this is to use a curated database source such as the NCBI RefSeq database, as used here, or the UniProt database. We also found here that inclusion of numerous closely related sequences does not provide any added value for the prediction of sites that are likely to be functionally significant (Figure S1 and Table S3). Rather, it is more useful to choose a broadly representative set of sequences that spans the full evolutionary depth of any given set of orthologs and to whatever extent possible equally populates the evolutionary lineages across this depth. There are numerous alignment programs that are available and the performance of these programs may differ according to the evolutionary relatedness of the sequences being analyzed. We recommend the initial use of several different multiple alignment programs followed by evaluation of the relative quality of the alignments produced. For example, the T-Coffee webserver provides for the use of numerous multiple sequence alignment programs in various combinations along with a tool for evaluation of alignment quality (http://tcoffee.crg.cat/). In addition, manual refinement of alignments can be particularly useful in generating the highest possible quality alignment. Finally, the results reported here suggest that several methods of site-specific evolutionary constraint may be of use, particularly PolyPhen2, ConSurf and ScoreCons. Thus, we would recommend using all of these methods and choosing sites that score high across all three as most likely to be functionally significant and thus the most viable targets for site-specific mutation experiments.

## Materials and Methods

### Quantifying Mutations in the CFTR Gene

The Cystic Fibrosis Mutation Database (http://www.genet.sickkids.on.ca/) was mined for clinically characterized CFTR coding sequence (*i.e.* exonic) mutations. CFTR coding sequence mutations were considered with respect to the amino acid changes they entail relative to the wild-type CFTR protein sequence (Genbank accession NP_000483.3). For each individual CFTR codon, all observed non-synonymous (*i.e.* amino acid altering) mutations (*a, b*) were scored based on the severity of the encoded amino acid change using the Grantham chemical difference matrix (*m*). The sum of scores (*S*) for each *codon* was then recorded: *S_codon_  =  Σ_over all substitutions_ m*(*a*,*b*). The codon mutation scores were scaled from 0 to 1 via normalization with the maximum score (*Max S_codon_*).

### CFTR Site-specific Evolutionary Parameters

CFTR amino acid and nucleotide coding sequences for a phylogenetically representative set of vertebrate species were taken from the NCBI Refseq database [29]: *Homo sapiens* (NP_000483, NM_000492), *Pan troglodytes* (NP_001073386, NM_001079917), *Pongo abelii* (NP_001162017, NM_001168545), *Macaca mulatta* (NP_001028110, NM_001032938), *Papio anubis* (NP_001106085, NM_001112615), *Equus caballus* (NP_001103980, NM_001110510), *Ovis aries* (NP_001009781, NM_001009781), *Bos taurus* (NP_776443, NM_174018), *Sus scrofa* (NP_001098420, NM_001104950), *Canis lupus familiaris* (NP_001007144, NM_001007143), *Felis catus* (NP_001041474, NM_001048009), *Oryctolagus cuniculus* (NP_001076185, NM_001082716), *Mus musculus* (NP_066388, NM_021050), *Rattus norvegicus* (NP_113694, NM_031506), *Ornithorhynchus anatinus* (NP_001229663, NM_001242734), *Gallus gallus* (NP_001099136, NM_001105666), *Salmo salar* (NP_001117005, NM_001123533), *Takifugu rubripes* (NP_001041505, NM_001048040), *Danio rerio* (NP_001038348, NM_001044883).

Amino acid and nucleotide multiple sequence alignments were created using the program ClustalW [24]. The resulting multiple sequence alignments were evaluated to calculate site-specific evolutionary constraint levels using six different approaches: SocreCons, ConSurf, PhastCons, DIVERGE and SIFT (using the amino acid alignment) as well as *Ka/Ks* (using the nucleotide coding sequence alignment). PolyPhen2 used the human CFTR amino acid sequence as a query in a homology search and created its own multiple sequence alignment. Descriptions for each of these measures, including conceptual information along with some details of how each is computed, can be found in Table 1.

For the six amino acid based methods, scores were scaled across the range from 0 to 1. To do this, site-specific evolutionary parameter scores were normalized with the maximum score. Scaling of ConSurf in this way necessitated a prior transformation step in order to make all values positive. To do this, all ConSurf parameter values were increased by the modulus of the minimum negative value. For SIFT scores, a transformation of the output probability values into expectation values was performed by multiplying the probability values for amino acid pairs with the corresponding values from the BLOSUM60 amino acid exchange matrix. *Ka/Ks* values were not scaled from 0 to 1 owing to the fact that they already fall into this range with the exception of 3 out of 670 codon values.

For all methods except DIVERGE and Polyphen2, these procedures were repeated over different evolutionary depths, *i.e.* using sets of sequences at different levels of evolutionary divergence from the human CFTR, as described in the Results and Discussion section and shown in Figure 1B. DIVERGE site-specific evolutionary constraint values were computed using the depth 4 (vertebrate) data set only. DIVERGE was run by splitting the vertebrate phylogeny at the deepest node separating the fish from the terrestrial vertebrates, and Type II divergence values were recorded. PolyPhen2 uses its own sequence similarity search and creates its own multiple sequence alignment to run, resulting in an equivalence to depth 4 (*i.e.* a maximum diversity of CFTR sequences included in the score calculation).

### Statistical Analyses

All statistical analyses were performed using the R package. Observed mutation and site-specific evolutionary score distributions were computed using density plots and for 20 discrete bins. Observed distributions were fit to their nearest theoretical distribution using maximum likelihood. Pairwise relationships, and their statistical significance, between the seven sets of site-specific evolutionary parameter values were evaluated with the Pearson correlation coefficient. Pearson correlation was also used to evaluate the pairwise relationships between the mutation scores and the site-specific evolution parameter values.

## Supporting Information

Figure S1 **Comparison of multiple sequence alignment methods.** The quality of multiple sequence alignments produced using different methods was inferred using average per site conservation scores based on the (A) ScoreCons algorithm (1 being highly conserved, 0 being highly divergent) or (B) the Core conservation score from the T-Coffee webserver (http://tcoffee.crg.cat/apps/tcoffee/do:core). Note that the T-Coffee algorithm was run in the default mode, which uses the T-Coffee algorithm alone, and in a custom mode using a combination of 5 different alignment algorithms (CLUSTALW, MAFFT, MUSCLE, ProbCons, T-Coffee).
(TIF)

Table S1 **Comparison of the strengths and weaknesses of the different site-specific evolutionary constraint methods used in the analysis.** The seven site-specific evolutionary constraint methods employed in the analysis with their respective strengths and weaknesses, if any.
(DOCX)

Table S2 **Effect of alignment method on the regression analysis.** The table depicts the effect of using different multiple sequence alignment methods on the Pearson Correlation Coefficient (PCC) and the *P*-value of the linear regression against the mutational score. * For PolyPhen2 and *Ka/Ks* (Selecton Server) the respective servers build their own alignment before computing the scores. PhastCons scores for this study were retrieved from UCSC Genome Browser. T-Coffee was run both on Default parameters (Def) and on multiple alignment algorithms (Custom). As evident, the regression values are not much different across multiple algorithms suggesting that they are not dependent on any particular alignment path or are an artifact of alignment quality.
(DOCX)

Table S3 **Effect of augmenting the sequence dataset.** The table presents how the different correlation gets affected from augmenting the sequence dataset with all the 192 available sequences in Genbank. The correlations were not found to be affected much with an expanded set of sequences. ND: Not Determined; NR: No Results.
(DOCX)
