# Supplementary figures and images for: Relating the Disease Mutation Spectrum to the Evolution of the Cystic Fibrosis Transmembrane Conductance Regulator (CFTR)
Source: PLoS One. 2012 Aug 7;7(8):e42336. doi: 10.1371/journal.pone.0042336 (PMC3413703; doi:10.1371/journal.pone.0042336)

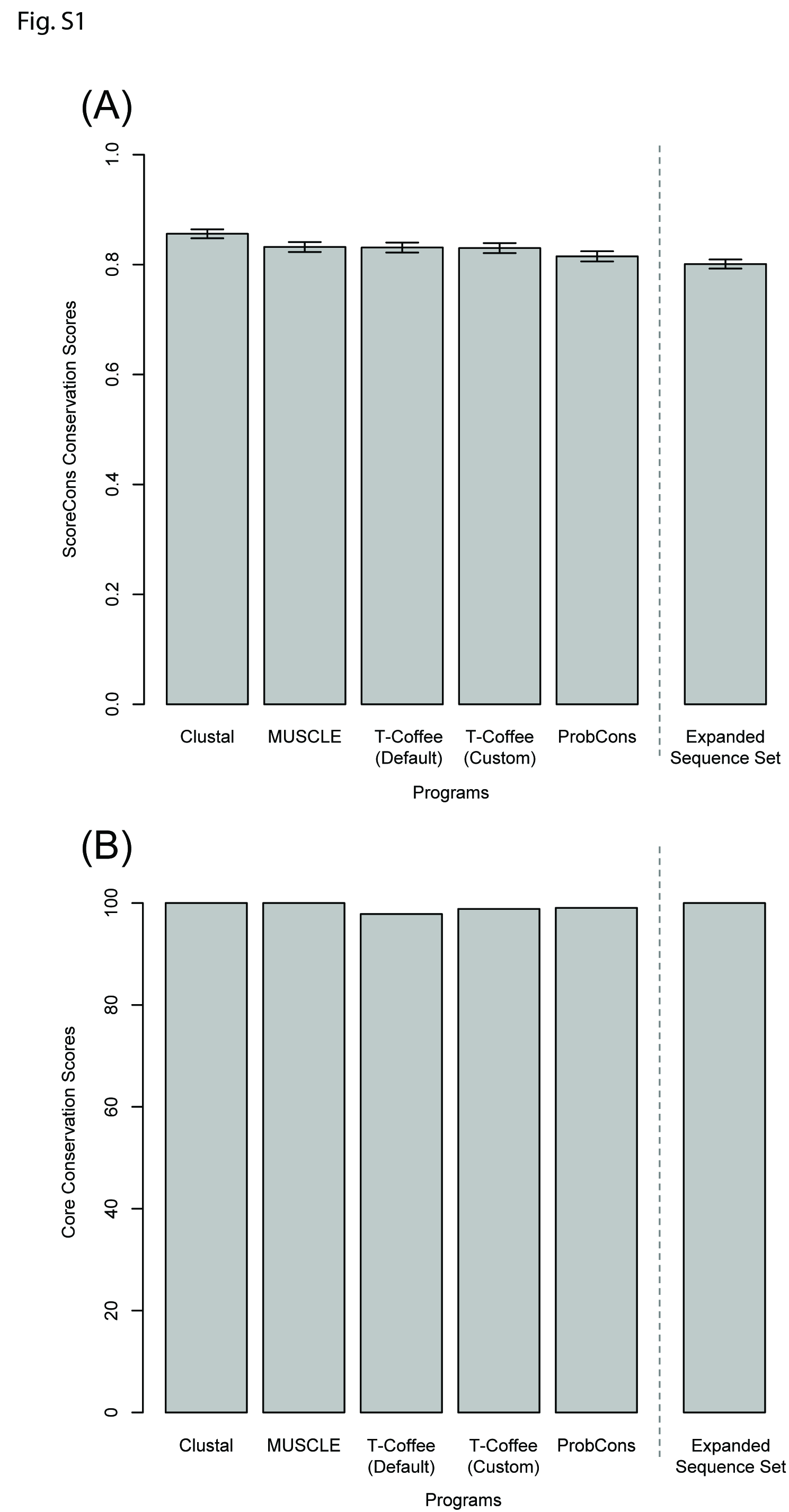

Supplement: Figure S1 — Comparison of multiple sequence alignment methods. The quality of multiple sequence alignments produced using different methods was inferred using average per site conservation scores based on the (A) ScoreCons algorithm (1 being highly conserved, 0 being highly divergent) or (B) the Core conservation score from the T-Coffee webserver (http://tcoffee.crg.cat/apps/tcoffee/do:core). Note that the T-Coffee algorithm was run in the default mode, which uses the T-Coffee algorithm alone, and in a custom mode using a combination of 5 different alignment algorithms (CLUSTALW, MAFFT, MUSCLE, ProbCons, T-Coffee). (TIF) [file pone.0042336.s001.tif]
