# Supplementary material for: Relating the Disease Mutation Spectrum to the Evolution of the Cystic Fibrosis Transmembrane Conductance Regulator (CFTR)
Source: PLoS One. 2012 Aug 7;7(8):e42336. doi: 10.1371/journal.pone.0042336 (PMC3413703; doi:10.1371/journal.pone.0042336)
Supplement: Table S1 — Comparison of the strengths and weaknesses of the different site-specific evolutionary constraint methods used in the analysis. The seven site-specific evolutionary constraint methods employed in the analysis with their respective strengths and weaknesses, if any. (DOCX) [file pone.0042336.s002.docx]

Table S1. **Comparison of the strengths and weaknesses of the different site-specific evolutionary constraint methods used in the analysis.** The seven site-specific evolutionary constraint methods employed in the analysis with their respective strengths and weaknesses, if any.

| **Parameter** | **Strength** | **Weakness** |
| --- | --- | --- |
| **(1) ScoreCons** | Straightforward conservation scoring scheme.  Quantify stereochemical diversity with a full substitution matrix.  Normalize against redundancy in the alignment  Penalizes gaps. | Does not take phylogeny into account, hence treats all sequences equally.  Uses a substitution matrix that relies solely on biochemical properties of the residues being compared. |
| **(2) ConSurf** | Quantify stereochemical diversity with a full substitution matrix.  Evolutionary rate computation by using either an empirical Bayesian Method or a Maximum Likelihood method.  Can correctly discriminate between conservation due to short evolutionary time and genuine sequence conservation. | Uses a substitution matrix that relies solely on biochemical properties of the residues being compared. |
| **(3) PhastCons** | Based on a phylogenetic hidden Markov model  Uses statistical models of nucleotide substitution that allow for multiple substitutions per site and for unequal rates of substitution between different pairs of bases. | Is primarily designed for calculating nucleotide conservation. |
| **(4) DIVERGE** | Takes into account the phylogenetic tree.  Computes coefficient of functional divergence between the two user-defined clusters. | Fails to compute on depths other than Vertebrata due to less number of sequences |
| **(5) PolyPhen2** | Performs its own sequence search and computes a set of sequence-based features which involve comparison of different properties of wild type and corresponding mutant allele.  Uses Naïve Bayes to predict functional importance of an allele replacement. | Alignment quality can not be manually verified. |
| **(6) SIFT** | Estimates probabilities that a given site belongs to the conserved site with the help of Dirichlet mixtures.  Quantify stereochemical diversity with a full substitution matrix. | Provides an n x 20 probability matrix (n = length of the amino acid), which requires further transformation to get a site-specific evolutionary score. |
| **(7) *Ka/Ks*** | A standard method for estimating the selective pressure at each site. | Designed for nucleotide sequences and Ks will become saturated for divergent sequences. |
