# Supplementary material for: Relating the Disease Mutation Spectrum to the Evolution of the Cystic Fibrosis Transmembrane Conductance Regulator (CFTR)
Source: PLoS One. 2012 Aug 7;7(8):e42336. doi: 10.1371/journal.pone.0042336 (PMC3413703; doi:10.1371/journal.pone.0042336)
Supplement: Table S2 — Effect of alignment method on the regression analysis. The table depicts the effect of using different multiple sequence alignment methods on the Pearson Correlation Coefficient (PCC) and the P-value of the linear regression against the mutational score. * For PolyPhen2 and Ka/Ks (Selecton Server) the respective servers build their own alignment before computing the scores. PhastCons scores for this study were retrieved from UCSC Genome Browser. T-Coffee was run both on Default parameters (Def) and on multiple alignment algorithms (Custom). As evident, the regression values are not much different across multiple algorithms suggesting that they are not dependent on any particular alignment path or are an artifact of alignment quality. (DOCX) [file pone.0042336.s003.docx]

Table S2. **Effect of alignment method on the regression analysis.** The table depicts the effect of using different multiple sequence alignment methods on the Pearson Correlation Coefficient (PCC) and the *P*-value of the linear regression against the mutational score.
* For PolyPhen2 and *Ka/Ks* (Selecton Server) the respective servers build their own alignment before computing the scores. PhastCons scores for this study were retrieved from UCSC Genome Browser. T-Coffee was run both on Default parameters (Def) and on multiple alignment algorithms (Custom). As evident, the regression values are not much different across multiple algorithms suggesting that they are not dependent on any particular alignment path or are an artifact of alignment quality.

| Alignment Program | ClustalW / Program Default | | | MUSCLE | | | T-Coffee (Def) | | | T-Coffee (Custom) | | | ProbCons | | |
| --- | --- | --- | --- | --- | --- | --- | --- | --- | --- | --- | --- | --- | --- | --- | --- |
| Evolutionary Method | **Rank** | **PCC** | ***P*-value** | **Rank** | **PCC** | ***P*-value** | **Rank** | **PCC** | ***P*-value** | **Rank** | **PCC** | ***P*-value** | **Rank** | **PCC** | ***P*-value** |
| PolyPhen2* | **1** | -0.191 | 1.44E-13 | **1** | -0.191 | 1.44E-13 | **1** | -0.191 | 1.44E-13 | **1** | -0.191 | 1.44E-13 | **1** | -0.191 | 1.44E-13 |
| ConSurf | **2** | -0.168 | 8.56E-11 | **4** | -0.139 | 7.11E-08 | **4** | -0.152 | 4.16E-09 | **4** | -0.150 | 7.15E-09 | **4** | -0.152 | 3.93E-09 |
| ScoreCons | **3** | -0.167 | 9.11E-11 | **2** | -0.174 | 1.69E-11 | **2** | -0.176 | 8.83E-12 | **2** | -0.174 | 1.62E-11 | **2** | -0.184 | 9.79E-13 |
| *Ka/Ks** | **4** | -0.165 | 1.77E-10 | **3** | -0.165 | 1.77E-10 | **3** | -0.165 | 1.77E-10 | **3** | -0.165 | 1.77E-10 | **3** | -0.165 | 1.77E-10 |
| SIFT | **5** | -0.107 | 3.82E-05 | **5** | -0.089 | 6.28E-04 | **5** | -0.090 | 5.21E-04 | **5** | -0.090 | 5.43E-04 | **5** | -0.090 | 5.38E-04 |
| PhastCons* | **6** | -0.105 | 5.33E-05 | **6** | -0.105 | 5.33E-05 | **6** | -0.105 | 5.33E-05 | **6** | -0.105 | 5.33E-05 | **6** | -0.105 | 5.33E-05 |
| DIVERGE | **7** | 0.015 | 5.62E-01 | **7** | 0.010 | 6.96E-01 | **7** | 0.036 | 1.68E-01 | **7** | 0.011 | 6.78E-01 | **7** | NR | NR |
