# Supplementary material for: Relating the Disease Mutation Spectrum to the Evolution of the Cystic Fibrosis Transmembrane Conductance Regulator (CFTR)
Source: PLoS One. 2012 Aug 7;7(8):e42336. doi: 10.1371/journal.pone.0042336 (PMC3413703; doi:10.1371/journal.pone.0042336)
Supplement: Table S3 — Effect of augmenting the sequence dataset. The table presents how the different correlation gets affected from augmenting the sequence dataset with all the 192 available sequences in Genbank. The correlations were not found to be affected much with an expanded set of sequences. ND: Not Determined; NR: No Results. (DOCX) [file pone.0042336.s004.docx]

Table S3. **Effect of augmenting the sequence dataset.** The table presents how the different correlation gets affected from augmenting the sequence dataset with all the 192 available sequences in Genbank. The correlations were not found to be affected much with an expanded set of sequences. ND: Not Determined; NR: No Results.

| Alignment Program | | Original Set | | Expanded Set | |
| --- | --- | --- | --- | --- | --- |
| Rank | **Method** | **PCC** | ***P*-value** | **PCC** | ***P*-value** |
| 1 | **PolyPhen2** | -0.191 | 1.44E-13 | -0.191 | 1.44E-13 |
| 2 | **ConSurf** | -0.168 | 8.56E-11 | -0.161 | 5.23E-10 |
| 3 | **ScoreCons** | -0.167 | 9.11E-11 | -0.200 | 8.80E-15 |
| 4 | ***Ka/Ks*** | -0.165 | 1.77E-10 | ND | ND |
| 5 | **SIFT** | -0.107 | 3.82E-05 | -0.025 | 3.34E-01 |
| 6 | **PhastCons** | -0.105 | 5.33E-05 | -0.105 | 5.33E-05 |
| 7 | **DIVERGE** | 0.015 | 5.62E-01 | NR | NR |
